# Mental Labyrinth: A Case of Treatment-Resistant Schizophrenia and Solutions

**DOI:** 10.1192/j.eurpsy.2025.900

**Published:** 2025-08-26

**Authors:** F. N. Meral, F. Ekici

**Affiliations:** 1 Psychiatry, Selcuk University, Konya, Türkiye

## Abstract

**Introduction:**

Despite advancements in the pharmacological treatment of schizophrenia, one-third of patients do not respond favorably. In many treatment algorithms, the lack of response after using two antipsychotic treatments at doses equivalent to 400–600 mg/day of chlorpromazine for four to six weeks is considered treatment-resistant schizophrenia.

**Objectives:**

This case report aims to examine the unresponsive symptoms in a case of treatment-resistant schizophrenia, the patient’s clinical course, and the approach to the treatment process.

**Methods:**

The hospitalized patient’s socio-demographic characteristics, medical and psychiatric history, and current complaints were examined in detail. Medications used, previous hospital visits, and admissions were evaluated.

**Results:**

A 34-year-old male was first hospitalized in 2016 during military service due to disorganized behaviors, preventing completion of service. A year later, noncompliance led to repeated hospitalizations for grandiose delusions and disorganized behaviors. Despite effective doses and durations of aripiprazole, risperidone, olanzapine, and paliperidone injection, his symptoms persisted. Upon presenting to us, his treatment was paliperidone 150 mg/month and risperidone 4 mg/day. Due to auditory hallucinations and persecutory and referential delusions, he was admitted for schizophrenia management. Clozapine was added per protocol, increased to 700 mg/day, then reduced to 500 mg/day due to anticholinergic side effects; weekly hemogram monitoring showed no agranulocytosis. Fluvoxamine was added for control and religious obsessions, increased to 300 mg/day. With partial symptom regression and ongoing resistance, eight sessions of electroconvulsive therapy were administered without reduction in psychotic symptoms. Observing benefits from typical antipsychotics, haloperidol loading doses of 50 mg, 150 mg, and 200 mg were given. After an 85-day hospitalization, significant improvement allowed discharge planning. He was discharged on clozapine 500 mg/day, fluvoxamine 300 mg/day, and haloperidol decanoate injection. Outpatient follow-ups showed remission and complete regression of psychotic symptoms.

**Conclusions:**

This case underscores the importance of treatment approaches in managing treatment-resistant schizophrenia. Formulating an effective treatment plan in such cases is often challenging and prolonged. As demonstrated, when initial treatments are inadequate, various steps are implemented per treatment protocols. In treatment-resistant patients, combining clozapine, ECT, and long-acting typical antipsychotics can effectively achieve long-term stabilization. The significance of regular follow-up, side effect management, and individualized treatment plans is evident in this case.

**Disclosure of Interest:**

None Declared

